# Evaluating use of two‐step International Ovarian Tumor Analysis strategy to classify adnexal masses identified in pregnancy: pilot study

**DOI:** 10.1002/uog.27707

**Published:** 2024-12-02

**Authors:** J. Barcroft, M. Pandrich, S. Del Forno, N. Cooper, K. Linton‐Reid, C. Landolfo, D. Timmerman, S. Saso, T. Bourne

**Affiliations:** ^1^ Department of Metabolism, Digestion and Reproduction Imperial College London London UK; ^2^ Department of Obstetrics and Gynaecology Queen Charlotte's and Chelsea Hospital, Imperial College Healthcare NHS Trust London UK; ^3^ Department of Gynaecology Hospital Policlinico S. Orsola – Malpighi Bologna Italy; ^4^ Department of Surgery and Cancer Imperial College London London UK; ^5^ Department of Development and Regeneration KU Leuven Leuven Belgium

**Keywords:** ADNEX model, benign simple descriptor, classification, ovarian cancer, ovarian cyst, pregnancy, ultrasonography, validation

## Abstract

**Objectives:**

The primary aim was to validate the International Ovarian Tumor Analysis (IOTA) benign simple descriptors (BDs) followed by the Assessment of Different NEoplasias in the adneXa (ADNEX) model, if BDs cannot be applied, in a two‐step strategy to classify adnexal masses identified during pregnancy. The secondary aim was to describe the natural history of adnexal masses during pregnancy.

**Methods:**

This was a retrospective analysis of prospectively collected data from women with an adnexal mass identified on ultrasonography during pregnancy between 2017 and 2022 at Queen Charlotte's and Chelsea Hospital, London, UK. Clinical and ultrasound data were extracted from medical records and ultrasound software. Adnexal masses were classified and managed according to expert subjective assessment (SA). Borderline ovarian tumors (BOTs) were classified as malignant. BDs were applied retrospectively to classify adnexal masses, and if BDs were not applicable, the ADNEX model (using a risk‐ of‐malignancy threshold ≥ 10%) was used, in a two‐step strategy. The reference standard was histology (where available) or expert SA at the postnatal ultrasound scan.

**Results:**

A total of 291 women with a median age of 33 (interquartile range (IQR), 29–36) years presented with an adnexal mass during pregnancy, at a median gestational age of 12 (IQR, 8–17) weeks. Of those, 267 (91.8%) were followed up to the postnatal period. Based on the reference standard, 4.1% (11/267) of adnexal masses were classified as malignant (all BOTs) and 95.9% (256/267) as benign. BDs were applicable in 68.9% (184/267) of adnexal masses; of these, only one (0.5%) BOT was misclassified as benign. The ADNEX model was used to classify the 83 residual masses and misclassified 3/10 (30.0%) BOTs as benign and 25/73 (34.2%) benign masses as malignant, of which 13/25 (52.0%) were classified as decidualized endometrioma on expert SA. The two‐step strategy had a specificity of 90.2%, sensitivity of 63.6%, negative predictive value of 98.3% and positive predictive value of 21.9%. A total of 56 (21.0%) women underwent surgical intervention: four (1.5%) as an emergency during pregnancy, four (1.5%) electively during Cesarean section and 48 (18.0%) postnatally. During follow‐up, 64 (24.0%) adnexal masses resolved spontaneously. Cyst‐related complications occurred in four (1.5%) women during pregnancy (ovarian torsion, *n* = 2; cyst rupture, *n* = 2) and six (2.2%) women in the postnatal period (all ovarian torsion). Overall, 196/267 (73.4%) women had a persistent adnexal mass at postnatal ultrasound. Presumed decidualization occurred in 31.1% (19/61) of endometriomas and had resolved in 89.5% (17/19) by the first postnatal ultrasound scan.

**Conclusions:**

BDs apply to most adnexal masses during pregnancy. However, the small number of malignant tumors in this cohort (4.1%) restricted the evaluation of the ADNEX model, so expert SA should be used to classify adnexal masses during pregnancy when BDs do not apply. A larger multicenter prospective study is required to evaluate the use of the ADNEX model to classify adnexal masses during pregnancy. Our data suggest that most adnexal masses can be managed expectantly during pregnancy, given the high rate of spontaneous resolution and low risk of complications. © 2024 The Author(s). *Ultrasound in Obstetrics & Gynecology* published by John Wiley & Sons Ltd on behalf of International Society of Ultrasound in Obstetrics and Gynecology.

## Abstract 2

This article's abstract has been translated into Spanish and Chinese. Follow the links from the abstract to view the translations.

## Abstract (es)

**RESUMEN:**

**Evaluación del uso de la estrategia internacional de análisis de tumores ováricos en dos pasos para clasificar masas anexiales identificadas en el embarazo: un estudio piloto**.

**Objetivos:**

El objetivo principal fue validar los descriptores benignos (DB) simples del Análisis Internacional de Tumores Ováricos (IOTA, por sus siglas en inglés) seguidos de la Evaluación de Diferentes Neoplasias en el modelo adneXa (ADNEX), si no se pueden aplicar los DB, en una estrategia de dos pasos para clasificar las masas anexiales identificadas durante el embarazo. El objetivo secundario fue describir el historial natural de las masas anexiales durante el embarazo.

**Métodos:**

Se realizó un análisis retrospectivo de datos recopilados prospectivamente de mujeres con una masa anexial identificada en la ecografía durante el embarazo entre 2017 y 2022 en el hospital Queen Charlotte's and Chelsea de Londres (Reino Unido). Los datos clínicos y ecográficos se obtuvieron de los historiales clínicos y del programa informático de ecografía. Las masas anexiales se clasificaron y trataron según la evaluación subjetiva (ES) por expertos. Los tumores ováricos limítrofes (TOL) se clasificaron como malignos. Los DB se aplicaron retrospectivamente para clasificar las masas anexiales, y si los DB no eran aplicables, se utilizó el modelo ADNEX (utilizando un umbral de riesgo de malignidad ≥10%), en una estrategia de dos pasos. El estándar de referencia fue la histología (cuando estaba disponible) o la ES por expertos en ecografía postnatal.

**Resultados:**

Un total de 291 mujeres con una mediana de edad de 33 años (rango intercuartílico [RIQ], 29–36) presentaron una masa anexial durante el embarazo, a una mediana de edad gestacional de 12 semanas (RIQ, 8–17). De ellas, a 267 (91,8%) se les dio seguimiento hasta el periodo postnatal. Según el estándar de referencia, el 4,1% (11/267) de las masas anexiales se clasificaron como malignas (todas TOL) y el 95,9% (256/267) como benignas. Los DB fueron aplicables al 68,9% (184/267) de las masas anexiales; de éstas, sólo un TOL (0,5%) se clasificó erróneamente como benigno. Se utilizó el modelo ADNEX para clasificar las 83 masas residuales y se clasificaron 3/10 (30,0%) TOL como benignas y 25/73 (34,2%) masas benignas como malignas, de las cuales 13/25 (52,0%) se clasificaron como endometrioma decidualizado en la ES por expertos. La estrategia en dos pasos tuvo una especificidad del 90,2%, una sensibilidad del 63,6%, un valor predictivo negativo del 98,3% y un valor predictivo positivo del 21,9%. Un total de 56 (21,0%) mujeres se sometieron a una intervención quirúrgica: cuatro (1,5%) como urgencia durante el embarazo, cuatro (1,5%) de forma electiva durante una cesárea y 48 (18,0%) en el posparto. Durante el seguimiento, 64 (24,0%) masas anexiales se resolvieron espontáneamente. Se produjeron complicaciones relacionadas con quistes en cuatro (1,5%) mujeres durante el embarazo (torsión ovárica, n=2; rotura de quiste, n=2) y en seis (2,2%) mujeres en el periodo postnatal (todas torsión ovárica). En total, 196/267 (73,4%) mujeres presentaban una masa anexial persistente en la ecografía postnatal. La presunta decidualización se produjo en el 31,1% (19/61) de los endometriomas y el 89,5% (17/19) se habían resuelto en la primera ecografía postnatal.

**Conclusiones:**

Los DB aplican a la mayoría de las masas anexiales durante el embarazo. Sin embargo, el escaso número de tumores malignos en esta cohorte (4,1%) restringió la evaluación del modelo ADNEX, por lo que cuando no apliquen los DB debería recurrirse a la ES por expertos para clasificar las masas anexiales durante el embarazo. Se requiere un estudio prospectivo multicéntrico más amplio para evaluar el uso del modelo ADNEX para clasificar las masas anexiales durante el embarazo. Los datos de este estudio sugieren que la mayoría de las masas anexiales pueden tratarse de forma expectante durante el embarazo, dada la alta tasa de resolución espontánea y el bajo riesgo de complicaciones.

## Abstract (zh)

**摘要:**

**评估使用国际卵巢肿瘤研究组两步策略对妊娠期发现的附件肿块进行分类:试点研究**

**目的:**

主要目的是先验证国际卵巢肿瘤研究组(IOTA)的良性简单描述符(BD)，如果无法应用BD，则使用多元附件肿瘤评估模型ADNEX，

分两步对妊娠期间发现的附件肿块进行分类。第二目的是描述妊娠期间附件肿块的自然病史。

**方法:**

本研究为对英国伦敦夏洛特女王和切尔西医院(Queen Charlotte's and Chelsea Hospital) 2017 年至 2022 年期间前瞻性收集的孕期超声检查发现附件肿块的妇女数据进行的回顾性分析。临床和超声数据均从医疗记录和超声软件中提取。附件肿块根据专家主观评估(SA)进行分类和管理。卵巢交界性肿瘤(BOT)被归类为恶性肿瘤。回顾性应用 BD 对附件肿块进行分类，如果 BD 不适用，则采用 ADNEX 模型(恶性风险阈值≥10%)，分两步进行。参考标准为组织学检查(如有)或产后超声扫描的专家SA。

**结果:**

共有 291 名中位数年龄为 33 岁(四分位距(IQR)为 29‐36 岁)的妇女在妊娠期间出现附件肿块，中位数孕周为 12 周(IQR 为 8‐17 周)。其中 267 人(91.8%)接受了产后随访。根据参考标准，4.1%(11/267)的附件肿块被归类为恶性(均为 BOT)，95.9%(256/267)被归类为良性。68.9%(184/267)的附件肿块适用 Bd；其中只有 1 例(0.5%)BOT 被误判为良性。使用 ADNEX 模型对剩余 83 例肿块进行分类，其中 3/10(30.0%)例 BOT 被归类为良性，25/73(34.2%)例良性肿块被归类为恶性，其中 13/25(52.0%)例根据专家 SA 被归类为蜕膜化子宫内膜异位。两步法的特异性为 90.2%，敏感性为 63.6%，阴性预测值为 98.3%，阳性预测值为 21.9%。共有 56 名(21.0%)妇女接受了手术治疗:其中 4 名(1.5%)是孕期急诊，4 名(1.5%)是剖宫产期间选择性治疗，48 名(18.0%)是产后治疗。在随访期间，64 例(24.0%)附件肿块自行消退。有 4 名(1.5%)妇女在孕期发生了与囊肿相关的并发症(卵巢扭转，2 例；囊肿破裂，2 例)，有 6 名(2.2%)妇女在产后发生了与囊肿相关的并发症(均为卵巢扭转)。总体而言，196/267(73.4%)名妇女在产后超声检查中发现有持续性附件肿块。31.1%(19/61)的子宫内膜异位症推断发生了蜕膜化，89.5%(17/19)的子宫内膜异位症在产后第一次超声检查时已经消退。

**结论:**

BD 适用于孕期的大多数附件肿块。然而，由于本队列中恶性肿瘤的数量较少(4.1%)，限制了对 ADNEX 模型的评估，因此当 BD 不适用时，应使用专家 SA 对孕期附件肿块进行分类。需要进行更大规模的多中心前瞻性研究，以评估使用 ADNEX 模型对孕期附件肿块进行分类的效果。我们的数据表明，鉴于附件肿块的自行消退率高且并发症风险低，大多数妊娠期附件肿块可作期待治疗。

**CONTRIBUTION**

*What are the novel findings of this work?*

This is the first study to validate the performance of the International Ovarian Tumor Analysis benign simple descriptors (BDs) and the Assessment of Different NEoplasias in the adneXa (ADNEX) model in a two‐step strategy to classify adnexal masses during pregnancy. BDs can be applied to most masses and perform well; however, evaluation of the ADNEX model was limited by the low malignancy rate (4.1%).

*What are the clinical implications of this work?*

BDs should be the first‐line approach to classify adnexal masses during pregnancy, and if BDs cannot be applied, expert subjective assessment should be used, rather than the ADNEX model. Expectantly managed adnexal masses in pregnant women have a low risk of cyst‐related complications and high chance of spontaneous resolution.

## INTRODUCTION

Adnexal masses are detected incidentally in 5.4–24.9% of pregnancies on first‐trimester ultrasonography^1^, ^2^. Most adnexal masses resolve spontaneously without intervention^1^, ^2^. The cumulative incidence of ovarian torsion in pregnant women with an adnexal mass is 3.0%^1^, which is higher than that reported over 2 years in a large premenopausal non‐pregnant cohort with an adnexal mass (0.5%)^3^. This difference may be attributable to the use of a clinical, rather than surgical, diagnosis of ovarian torsion during pregnancy^1^. Ovarian malignancy is rare in a low‐risk unselected pregnant population, with no case of invasive malignancy identified by either Condous *et al*.^1^ or Yazbek *et al*.^2^. However, in a high‐risk pregnant population with an adnexal mass referred to a tertiary oncology center, the incidence of invasive malignancy was found to be 14%^4^.

Expectant management of adnexal masses is preferable in asymptomatic pregnant women, given the maternal and fetal risks associated with surgery, including preterm delivery and fetal growth restriction^5^, ^6^. Surgery is indicated in suspected cyst‐related complications or invasive malignancy^4^, ^6^, ^7^, ^8^. Pregnancy can induce morphological changes within endometriomas, a process known as decidualization^9^, ^10^, ^11^, ^12^, which may share ultrasound features with both borderline ovarian tumor (BOT) and early‐stage invasive ovarian cancer, making the classification of adnexal masses more difficult during pregnancy^9^, ^13^, ^14^, ^15^, ^16^. Safe expectant management relies on accurate ultrasound classification of the adnexal masses. The International Ovarian Tumor Analysis (IOTA) group identified four benign simple descriptors (BDs)^17^ and developed ultrasound‐based models to support the classification of adnexal masses by less experienced ultrasound examiners, including the Assessment of Different NEoplasias in the adneXa (ADNEX) model^18^, ^19^. IOTA models have been validated widely outside of pregnancy^19^, ^20^, ^21^, ^22^, ^23^, but only three retrospective studies have validated them with respect to high‐risk masses during pregnancy^24^, ^25^, ^26^.

In the two‐step strategy, the BDs are used initially to classify an adnexal mass, and if the BDs cannot be applied, the ADNEX model is employed^17^, ^18^. The two‐step strategy accurately classified adnexal masses in a large non‐pregnant cohort (*n* = 4905; area under the receiver‐operating‐characteristics curve, 0.95), and the risk of malignancy was low (< 1%) when the BDs were applicable^17^.

The primary aim of this study was to validate the IOTA two‐step strategy for classifying adnexal masses as benign or malignant during pregnancy. The secondary aim was to describe the natural history of adnexal masses during pregnancy.

## METHODS

This was a retrospective analysis of prospectively collected data from women presenting with a probable non‐physiological adnexal mass identified on ultrasound during pregnancy between March 2017 and August 2022 at Queen Charlotte's and Chelsea Hospital (QCCH), a University Teaching Hospital in London, UK, with a high‐risk obstetric population. QCCH is a gynecological oncology center, with a specialist ovarian cyst clinic led by expert ultrasound examiners. The ovarian cyst clinic receives referrals for patients with probable non‐physiological adnexal masses found at the time of dating ultrasound (11–14‐week scan) and from the early‐pregnancy assessment unit, local obstetric units (without the ultrasound expertise to follow‐up adnexal masses) and primary care. Women underwent a transvaginal and/or transabdominal ultrasound scan (vaginal probe frequencies of 4–7 MHz) using Samsung WS80 or W10 (Samsung Medison, Seoul, Republic of Korea) and GE Voluson E10 (GE Healthcare, Zipf, Austria) ultrasound machines. Scans were performed by Level‐1 and Level‐2 ultrasound operators, according to European Federation of Societies for Ultrasound in Medicine and Biology (EFSUMB) criteria, who were trained in IOTA terminology and received expert supervision^27^.

Eligibility criteria were pregnant women aged ≥ 18 years with a non‐physiological adnexal mass ≥ 1 cm on ultrasonography. Exclusion criteria were incomplete ultrasound data and age < 18 years.

Adnexal masses were classified according to expert subjective assessment (SA) at the time of ultrasound examination. BOTs were classified as malignant. Ultrasound and clinical data were recorded prospectively within the Astraia database (Nexus/Astraia, Ismaning, Germany) as part of routine care. The standardized ultrasound report listed the type of lesion, cyst content, cyst wall regularity, lesion diameter, number of locules, presence of acoustic shadows and ascites, diameter of any solid components, and papillary projections (size and presence of flow). Clinical data were extracted from medical records, including age, parity, gestational age, symptoms (pain and/or bleeding), mode of conception, pregnancy outcome and cyst‐related complications. BDs were applied retrospectively as the first‐line approach to classify adnexal masses^17^, ^18^. If BDs were not applicable, the ADNEX score (using a 10% risk‐of‐malignancy threshold) was calculated retrospectively and used to classify the residual adnexal masses in a two‐step strategy. In cases of bilateral adnexal masses, the most complex or largest mass was used in the study.

Ultrasound surveillance during pregnancy was tailored to the individual. Symptomatic women and those with an adnexal mass exhibiting suspicious ultrasound features were kept under close ultrasonographic surveillance during pregnancy to identify features of invasive cancer. Asymptomatic women and those with an adnexal mass classified as benign based on expert SA underwent a routine postnatal ultrasound examination, ideally between 6 and 12 weeks after delivery.

The primary outcome was to validate the IOTA two‐step strategy for classifying adnexal masses during pregnancy. The reference standard was expert SA at the first postnatal ultrasound scan or, in women with a benign adnexal mass who underwent surgery and all women with a malignant adnexal mass, histological analysis. Secondary outcomes included the morphological changes observed in adnexal masses during pregnancy, focusing on presumed decidualization, and the incidence of cyst‐related complications (ovarian torsion and cyst rupture).

The management of adnexal masses during pregnancy was registered as an audit within the Imperial College Healthcare NHS Trust (GRM_034 ‐ ADNEXAL MASSES IN PREGNANCY) to permit data extraction; thus, formal ethical approval was not required.

Data were collected in an Excel file (Microsoft, Redmond, WA, USA) and presented as median (interquartile range (IQR)) or *n* (%). The diagnostic performance of the two‐step strategy was summarized as sensitivity, specificity and positive and negative predictive values, which were calculated using the Python Package Index (Python Software Foundation, Wilmington, DE, USA; https://pypi.org/).

## RESULTS

**Figure 1:**
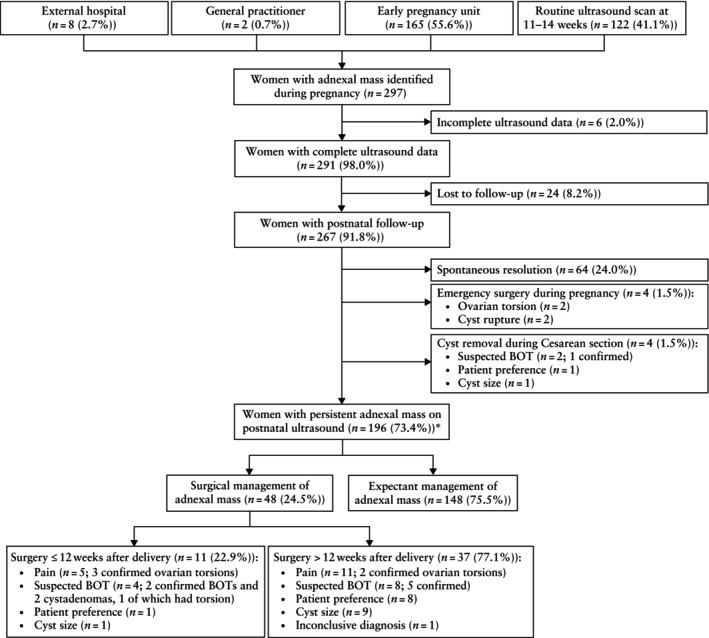
Flowchart summarizing inclusion and outcome of women with adnexal mass during pregnancy. *One woman underwent laparoscopic detorsion (not a cystectomy) during pregnancy, had a persistent adnexal mass on postnatal ultrasound and remains in conservative follow‐up. BOT, borderline ovarian tumor.

During the period between March 2017 and August 2022, 297 pregnant women were referred with an adnexal mass: 165/297 (55.6%) were referred from the early‐pregnancy assessment unit, 122/297 (41.1%) following a routine first‐trimester scan, 2/297 (0.7%) from primary care and 8/297 (2.7%) from local obstetric units (Figure 1). Of these, six (2.0%) women were excluded due to incomplete ultrasound data, resulting in a cohort of 291 women (median age, 33 (IQR, 29–36) years) (Table 1). At the antenatal (inclusion) ultrasound scan, 191/291 (65.6%) adnexal masses were visualized on transvaginal ultrasonography and 100/291 (34.4%) could only be seen transabdominally. Of the 291 women, 72 (24.7%) had a known adnexal mass before pregnancy and 26 (8.9%) women had bilateral adnexal masses. The median gestational age at the inclusion ultrasound scan was 12 (IQR, 8–17) weeks. Most women conceived spontaneously (276/291 (94.8%)) and were asymptomatic (189/291 (64.9%)). Overall, 267/291 (91.8%) women were followed up to the postnatal period and 24/291 (8.2%) were lost to follow‐up. The ultrasound features of the adnexal masses are outlined in Table 2.

**Table 1:** Clinical characteristics of 291 women with adnexal mass identified during pregnancy

Characteristic	Value
Age (years)	33 (29–36)
GA at inclusion US scan (weeks)	12 (8–17)
Parity	
0	180 (61.9)
1	82 (28.2)
2	20 (6.9)
3	4 (1.4)
> 3	5 (1.7)
Mode of conception	
Spontaneous	276 (94.8)
ART	15 (5.2)
Symptoms	
Pain only	68 (23.4)
Bleeding only	18 (6.2)
Pain and bleeding	16 (5.5)
No symptoms	189 (64.9)
Pregnancy outcome	
Vaginal delivery	148 (50.9)
Cesarean section	69 (23.7)
Miscarriage	56 (19.2)
Termination	8 (2.7)
Ectopic	2 (0.7)
Unknown	8 (2.7)

Data are given as median (interquartile range) or *n* (%). ART, assisted reproductive technology; GA, gestational age; US, ultrasound.

**Table 2:** Features of adnexal masses observed on antenatal and postnatal ultrasound scans

Feature	Antenatal (*n* = 291)	Postnatal (*n* = 196)
Maximum diameter of lesion (mm)	48 (34–65.5)	42.5 (26–63)
Maximum diameter of solid component (mm)	14.5 (11–21.5)	13 (7–18)
Expert SA		
Benign	277 (95.2)	187 (95.4)
Malignant	13 (4.5)	8 (4.1)
Inconclusive	1 (0.3)	1 (0.5)
Laterality		
Unilateral	265 (91.1)	170 (86.7)
Bilateral	26 (8.9)	26 (13.3)
Acoustic shadows	92 (31.6)	92 (46.9)
Cyst content		
Anechoic	126 (43.3)	53 (27.0)
Low‐level	22 (7.6)	18 (9.2)
Ground‐glass	58 (19.9)	54 (27.6)
Mixed	68 (23.4)	63 (32.1)
Hemorrhagic	9 (3.1)	3 (1.5)
N/A (solid)	8 (2.7)	5 (2.6)
Doppler color score		
1	239 (82.1)	163 (83.2)
2	45 (15.5)	30 (15.3)
3	4 (1.4)	3 (1.5)
4	3 (1.0)	0 (0)
Irregular cyst wall	99 (34.0)	79 (40.3)
Incomplete septa	8 (2.7)	6 (3.1)
Lesion type		
Unilocular	218 (74.9)	150 (76.5)
Unilocular solid	27 (9.3)	13 (6.6)
Multilocular	29 (10.0)	20 (10.2)
Multilocular solid	9 (3.1)	8 (4.1)
Solid	8 (2.7)	5 (2.6)
Number of locules		
1	246 (84.5)	158 (80.6)
2	14 (4.8)	17 (8.7)
3	12 (4.1)	3 (1.5)
4	3 (1.0)	3 (1.5)
5–10	9 (3.1)	10 (5.1)
> 10	2 (0.7)	1 (0.5)
N/A	5 (1.7)	4 (2.0)
Papillary projections	31 (10.7)	18 (9.2)
Number of projections		
1	20/31 (64.5)	13/18 (72.2)
2	5/31 (16.1)	3/18 (16.7)
3	2/31 (6.5)	0/18 (0)
> 3	4/31 (12.9)	2/18 (11.1)
Papillary flow present	10/31 (32.3)	6/18 (33.3)
Suggestion of decidualization within endometrioma, based on expert SA	19/61 (31.1)	1/55 (1.8)

Data are given as median (interquartile range), *n* (%) or *n*/*N* (%).

N/A, not applicable; SA, subjective assessment.

### Two‐step strategy

Based on the reference standard, 4.1% (11/267) of adnexal masses were classified as malignant (all BOTs) and 95.9% (256/267) as benign (41 based on histology and 215 based on expert SA). Only 1/11 (9.1%) malignant mass was referred from an external site (primary care or local obstetric unit) (Table 3). BDs were applicable in 184/267 (68.9%) adnexal masses. The presumed ultrasound‐based diagnoses were as follows: endometrioma (BD1, *n* = 41 (22.3%)); dermoid (BD2, *n* = 51 (27.7%)); simple cyst/cystadenoma (BD3, *n* = 80 (43.5%)); and other unilocular cysts (BD4, *n* = 12 (6.5%)) (Table 4). BDs classified correctly 183/184 (99.5%) adnexal masses. BDs misclassified 1/184 (0.5%) BOT as a benign adnexal mass (Figure S1).

**Table 3:** Ultrasound (US) features and classification of 11 histologically confirmed malignant adnexal masses

								Classification at antenatal US				Classification at postnatal US		
Case	External referral	Maximum diameter of lesion (mm)	Maximum diameter of solid component (mm)	Presence of > 10 locules	Papillary projections (*n*)	Acoustic shadows present	Ascites present	Expert SA	ADNEX (risk‐of‐ malignancy score)*	Mass visualized on antenatal TVS	GA at inclusion US (weeks)	Expert SA	ADNEX (risk‐of‐ malignancy score)*	Histology
1	No	88	17	No	4	No	No	BOT (malignant)	Malignant (58.7%)	No	25	N/A‡	N/A‡	Serous BOT
100	No	20	15	No	4	No	No	BOT (malignant)	Malignant (69.4%)	Yes	4	Malignant	Malignant (67.8%)	Serous BOT
123	No	83	N/A	No	0	No	No	Mucinous cystadenoma (benign)	Benign (4.0%)†	No	12	Benign	Benign (0.5%)	Mucinous BOT
225	No	27	26	No	1	No	No	BOT (malignant)	Malignant (45.9%)	Yes	12	Malignant	Malignant (59.1%)	Seromucinous BOT
231	No	19	13	No	1	No	No	Decidualized endometrioma (benign)	Malignant (34.3%)	Yes	13	Malignant	Benign (7.6%)	Serous BOT
238	No	46	20	No	> 4	No	No	BOT (malignant)	Malignant (72.2%)	No	20	Malignant	Malignant (71.1%)	Serous BOT
247	Yes	75	28	No	2	No	No	BOT (malignant)	Malignant (56.3%)	No	32	Malignant	Malignant (62.0%)	Serous BOT
250	No	119	N/A	No	0	No	No	Cystadenoma (benign)	Benign (4.8%)	No	13	Benign	Benign (8.5%)	Mucinous BOT
252	No	166	N/A	No	0	Yes	No	Cystadenoma (benign)	Benign (0.9%)	No	32	Malignant	Malignant (17.7%)	Mucinous BOT
286	No	39	15	No	3	No	No	BOT (malignant)	Malignant (53.1%)	Yes	10	Malignant	Malignant (11.6%)	Serous BOT
288	No	131	N/A	No	0	Yes	No	Cystadenofibroma (benign)	Benign (0.7%)	No	18	N/A§	N/A§	Mucinous BOT

* Without CA125 and using a 10% risk‐of‐malignancy threshold to define malignancy.

† Benign descriptor 4 applied.

‡ Removed at Cesarean section.

§ Removed on postnatal day 2.

ADNEX, Assessment of Different NEoplasias in the adneXa; BOT, borderline ovarian tumor; GA, gestational age; N/A, not applicable; SA, subjective assessment; TVS, transvaginal ultrasound.

**Table 4:** Performance of benign simple descriptors (BDs) for classification of adnexal masses in 267 pregnant women

BD	*n*	Benign*	Malignant†
Any	184	183/184 (99.5)	1/184 (0.5)
BD1	41	41/41 (100)	0/41 (0)
BD2	51	51/51 (100)	0/51 (0)
BD3	80	80/80 (100)	0/80 (0)
BD4	12	11/12 (91.7)	1/12 (8.3)

Data are given as *n* or *n*/*N* (%).

* Based on histology or expert subjective assessment.

† Based on histology.

BD1, unilocular cyst with ground‐glass echogenicity and largest diameter < 10 cm in premenopausal woman; BD2, unilocular cyst with mixed echogenicity, acoustic shadows and largest diameter < 10 cm in premenopausal woman; BD3, unilocular cyst with anechoic cyst fluid, smooth internal walls and largest diameter < 10 cm; BD4, all other unilocular cysts with smooth internal walls and largest diameter < 10 cm.

Using a 10% risk‐of‐malignancy threshold, the ADNEX model was applied to the 83 (31.1%) residual adnexal masses for which the BDs were not applicable, in a two‐step strategy (Table 5). Using the reference standard, the ADNEX model classified incorrectly 28/83 (33.7%) residual adnexal masses: three (3/10 (30.0%)) BOTs were classified as benign and 25 (25/73 (34.2%)) benign adnexal masses were classified as malignant (Tables 5 and S1). Of the 25 benign adnexal masses that were misclassified as malignant by the ADNEX model, 21/25 (84.0%) had at least one papillary projection and 13/25 (52.0%) were presumed to be decidualized endometriomas (based on expert SA), with confirmed resolution of decidualization in the postnatal period (Table S2). Overall, the two‐step strategy incorrectly classified 25/256 (9.8%) benign masses as malignant and 4/11 (36.4%) BOTs as benign (Table 5). When considering only those adnexal masses classified on transvaginal ultrasonography (177/267), which included four malignant adnexal masses, the two‐step strategy did not misclassify any malignant cases. The two‐step strategy had a specificity of 90.2% (95% CI, 86.6–93.9%), sensitivity of 63.6% (95% CI, 35.2–92.1%), negative predictive value of 98.3% (95% CI, 96.6–100%) and positive predictive value of 21.9% (95% CI, 7.6–36.2%).

**Table 5:** Performance of benign simple descriptors (BDs), when applicable, and Assessment of Different NEoplasia of the adneXa (ADNEX) model, when BDs could not be applied, for classification of adnexal masses in 267 pregnant women

Method	All (*n* = 267)	Benign* (*n* = 256)	Malignant† (*n* = 11)
BDs	184	183	1
ADNEX model	83	73	10
Benign‡	51	48	3
Malignant§	32	25	7

Data are given as *n*.

* Based on histology or expert subjective assessment.

† Based on histology.

‡ Risk of malignancy < 10%.

§ Risk of malignancy ≥ 10%.

### Cyst outcome

Spontaneous resolution occurred in 64/267 (24.0%) women, while 56/267 (21.0%) women required surgical intervention: four (1.5%) as an emergency during pregnancy, four (1.5%) at delivery and 48 (18.0%) in the postnatal period, of which 11 (4.1%) occurred within the first 12 weeks and 37 (13.9%) occurred > 12 weeks after delivery (Table 6, Figure 1). Cyst‐related complications occurred in four (1.5%) women during pregnancy (ovarian torsion, *n* = 2; cyst rupture, *n* = 2) and in six (2.2%) cases in the postnatal period (ovarian torsion, *n* = 6). A total of 196 (73.4%) women had a persistent adnexal mass on postnatal ultrasound, including one woman who experienced ovarian torsion during pregnancy and underwent detorsion.

**Table 6:** Clinical details of 56 women who underwent surgical intervention for adnexal mass

Characteristic	Value
Timing of surgery	
First trimester	2 (3.6)
Second trimester	1 (1.8)
Third trimester	1 (1.8)
At delivery	4 (7.1)
≤ 12 weeks after delivery	11 (19.6)
> 12 weeks after delivery	37 (66.1)
Surgery	
Emergency	11 (19.6)
Elective	45 (80.4)
Cyst‐related complications	
Ovarian torsion	8 (14.3)
Cyst rupture	2 (3.6)
No complications	46 (82.1)
Histology	
Malignant (borderline)	11 (19.6)
Serous borderline	6/11 (54.5)
Seromucinous borderline	1/11 (9.1)
Mucinous borderline	4/11 (36.4)
Benign	41 (73.2)
Mature teratoma	22/41 (53.7)
Benign cystadenoma	12/41 (29.3)
Struma ovarii	2/41 (4.9)
Hydrosalpinx/fimbrial	2/41 (4.9)
Ectopic pregnancy*	1/41 (2.4)
Corpus albicans	1/41 (2.4)
Fibroma	1/41 (2.4)
No histology†	4 (7.1)
Pregnancy outcome	
Vaginal delivery	33 (58.9)
Cesarean section	13 (23.2)
Miscarriage	6 (10.7)
Termination of pregnancy	4 (7.1)
Stage (if malignant)	
IA	6/11 (54.5)
IB	0/11 (0)
IC‡	5/11 (45.5)

Data are given as *n* (%) or *n*/*N* (%).

* A persistent mass in the Fallopian tube in a patient with a negative serum human chorionic gonadotropin test following termination of pregnancy was managed surgically and confirmed to be an ectopic pregnancy on histology.

† Histology was not available for cases of ovarian torsion/cyst rupture during pregnancy.

‡ Two Stage‐IC cases were due to intraoperative spill.

During pregnancy, the two (0.75%) cases of ovarian torsion occurred, respectively, at 9 weeks in a hemorrhagic cyst and 15 weeks in a benign cystadenoma (Table 7). Both were successfully detorted laparoscopically and neither required an oophorectomy nor experienced recurrent torsion. The two (0.75%) cases of cyst rupture occurred in mature teratomas, at 11 weeks (open cystectomy) and 30 weeks (laparoscopic washout), respectively. All four women operated on during pregnancy delivered at term, with no significant complications. No adnexal mass was removed electively during pregnancy. At Cesarean section (obstetric indication), four (1.5%) women underwent cyst removal, of which two were because of a suspected BOT (one had confirmed BOT on histology), one was because of the size of the cyst (benign histology) and one was due to patient preference (benign histology).

**Table 7:** Characteristics of 19 adnexal masses managed surgically during pregnancy, delivery or the immediate postnatal period (≤ 12 weeks after delivery)

Timing of surgery	Surgical procedure	Indication	Ultrasound diagnosis	Maximum cyst diameter (mm)	Complication	Histology	Pregnancy outcome
Antenatal							
9 weeks	Laparoscopic ovarian detorsion	Suspected torsion	Hemorrhagic	49	Torsion	N/A	SVD
11 weeks	Open ovarian cystectomy	Suspected torsion	Dermoid	105	Cyst rupture	Mature teratoma	SVD
15 weeks	Laparoscopic ovarian detorsion	Suspected torsion	Benign cystadenoma	58	Torsion	N/A*	ElCS
30 weeks	Laparoscopic appendicectomy + pelvic washout†	Suspected appendicitis	Dermoid	108	Cyst rupture	Mature teratoma	AVD
At delivery							
37 weeks	Open cystectomy	Patient preference	Mucinous cystadenoma	110	None	Mucinous cystadenoma	ElCS
38 weeks	Open bilateral ovarian cystectomy	Suspected BOT	Serous BOT	88	None	Serous BOT	ElCS
38 weeks	Open unilateral salpingo‐oophorectomy	Suspected BOT	Mucinous cystadenoma (cannot exclude BOT)	99	None	Mucinous cystadenoma	ElCS
39 weeks	Open cystectomy	Cyst size	Cystadenofibroma	116	None	Struma ovarii	ElCS
Postnatal							
< 1 week	Open unilateral salpingo‐oophorectomy	Suspected torsion	Mucinous cystadenofibroma	131	Torsion	Mucinous BOT	AVD
3 weeks	Laparoscopic ovarian detorsion + cystectomy	Suspected torsion	Dermoid	111	Torsion	Mature cystic teratoma	SVD
4 weeks	Open unilateral salpingo‐oophorectomy	Suspected BOT	Mucinous cystadenoma (cannot exclude BOT)	203	Torsion	Mucinous cystadenoma	AVD
5 weeks	Laparoscopic ovarian cystectomy	Pain	Dermoid	71	None	Mature teratoma	Miscarriage
8 weeks	Diagnostic laparoscopy	Pain	Paraovarian cystadenoma	121	None	N/A	Miscarriage
8 weeks	Laparoscopic ovarian cystectomy + omental biopsy	Suspected BOT	Seromucinous BOT	30	None	Seromucinous BOT	EmCS (36 weeks)
8 weeks	Laparoscopic ovarian cystectomy	Patient preference	Dermoid	64	None	Dermoid	Miscarriage
12 weeks	Laparoscopic paraovarian cystectomy	Suspected BOT	Paraovarian cystadenofibroma (cannot exclude BOT)	26	None	Serous cystadenoma	Miscarriage
12 weeks	Laparoscopic ovarian cystectomy	Pain	Cystadenofibroma	78	Torsion	Mucinous cystadenoma	AVD
12 weeks	Open cystectomy	Cyst size	Dermoid	150	None	Dermoid	TOP
12 weeks	Open unilateral salpingo‐ophorectomy	Suspected BOT	Mucinous BOT	173	None	Mucinous BOT	SVD

* Cyst was still present on postnatal ultrasound assessment (29 mm) and patient remains in conservative follow‐up.

† Laparoscopic cystectomy performed postnatally.

AVD, assisted vaginal delivery; BOT, borderline ovarian tumor; ElCS, elective Cesarean section; EmCS, emergency Cesarean section; N/A, not applicable; SVD, spontaneous vaginal delivery; TOP, termination of pregnancy.

Up to 12 weeks postnatally, 11/267 (4.1%) women underwent surgery, of which five were due to pain (three were confirmed ovarian torsions), four were for a suspected BOT (two were confirmed as such on histology and two were benign cystadenomas, one of which had torsion), one was due to patient preference (benign histology) and one was for cyst size (benign histology) (Figure 1). A further 37/267 (13.9%) women underwent surgical intervention up to 36 months postnatally (Table 6, Figure 1); eight were for a suspected BOT, of which five were confirmed on histology.

### Decidualization

Based on expert SA, presumed decidualization was noted in 19/61 (31.1%) endometriomas at the inclusion ultrasound scan. Most (12/19 (63.2%)) women with a presumed decidualized endometrioma had a prepregnancy ultrasound scan that confirmed the presence of an endometrioma. The ultrasound features of decidualized endometriomas are summarized in Table S2. Most (12/19 (63.2%)) presumed decidualized endometriomas were classified as unilocular solid masses. At least one papillary projection was present in 15/19 (78.9%) masses and irregular cyst walls were present in 18/19 (94.7%). Evidence of decidualization had resolved by the third trimester in one (5.3%) patient, by the first postnatal ultrasound scan in 16 (84.2%) patients (Figure S2) and by 17 months postnatally in one (5.3%) patient (Figure S3). One presumed decidualized endometrioma was reclassified as a serous BOT at the postnatal scan, which was confirmed on histology (Figure S4). Of the 18 confirmed decidualized endometriomas with resolution in the postnatal period, the ADNEX model misclassified 13 (72.2%) as malignant (Table S1).

## DISCUSSION

In our study population, we found that BDs applied to most adnexal masses during pregnancy, and that the risk of malignancy was very low (≤ 0.5%) when BDs were applicable. BDs, when applied, misclassified only one BOT, while the ADNEX model misclassified three BOTs using the two‐step strategy. The two‐step strategy had excellent specificity (90.2%) but poor sensitivity (63.6%). If only cases examined using transvaginal ultrasonography are considered, no BOTs were misclassified by the two‐step strategy, which highlights the additional challenge of assessing adnexal masses during pregnancy when visualization is possible using only transabdominal ultrasonography.

BDs were applicable in 68.9% of adnexal masses in this relatively low‐risk population, compared with 37% in the large non‐pregnant cohort (*n* = 4905) described by Landolfo *et al*.^17^. This difference is to be expected, given that the non‐pregnant cohort was older (median, 48 *vs* 33 years) and included only adnexal masses that were persistent at 12‐month follow‐up, therefore effectively removing a proportion of simple/functional cysts (which could be classified by BDs) that had resolved during follow‐up. The low risk of malignancy in adnexal masses (0.5%) observed in the present study in which BDs were applicable was comparable to that in the non‐pregnant cohort (0.7%)^17^, and supports the use of BDs as the first‐line approach to classify adnexal masses during pregnancy.

The presence of decidualization likely contributed to the poor performance of the ADNEX model during pregnancy, given that 13/25 (52.0%) benign masses misclassified by the ADNEX model were decidualized endometriomas, with confirmed resolution in the postnatal period. The diagnostic challenge posed by decidualization during pregnancy was also demonstrated by Testa *et al*., who described the removal of five presumed BOTs during pregnancy, which were confirmed as decidualized endometriomas on histology^4^. Deferring management decisions to the postnatal period, when appropriate, would allow for the resolution of decidualization and reassessment of the adnexal mass using transvaginal ultrasonography, which is preferable given that the ADNEX model was developed and validated on transvaginal ultrasound and is known to perform well outside of pregnancy^18^, ^19^, ^28^. While all cases of BOT were managed expectantly during pregnancy, with no invasive cancers in this cohort, the small number of malignancies (*n* = 11 (4.1%)) limited the conclusions that could be drawn on the safety of an expectant management approach for suspected BOT during pregnancy.

The low malignancy rate in this cohort also restricted the evaluation of the ADNEX model as part of the two‐step strategy. A larger, prospective, multicenter study that is adequately powered is required to validate the use of IOTA diagnostic models during pregnancy to support the classification and management of adnexal masses by less experienced ultrasound examiners. Until the ADNEX model has been validated in pregnancy, we recommend using expert SA to classify adnexal masses for which the BDs cannot be applied.

Previous studies have evaluated the performance of the ADNEX model during pregnancy. Lee *et al*. applied retrospectively the ADNEX model (using a 14% risk‐of‐malignancy cut‐off) in a cohort of 236 pregnant women who underwent surgery for an adnexal mass, and reported a specificity of 84.8% and a sensitivity of 61.5%^24^. Their results were limited by the exclusion of BOTs and the retrospective extraction of ultrasound features and application of the ADNEX model^24^. Czekierdowski *et al*. described the prospective application of the ADNEX model (using a 20% risk‐of‐malignancy cut‐off) to adnexal masses managed surgically, but their conclusions were limited by the small sample size (*n* = 36)^25^. We reported the sensitivity and specificity of the two‐step strategy, rather than that of the ADNEX model alone, given the small number of cases to which the ADNEX model was applied (*n* = 83) and the limitations of using these diagnostic performance metrics in small, low‐risk datasets containing only a few malignant cases.

The incidence of ovarian torsion during pregnancy (0.7%) is slightly higher than the cumulative 2‐year incidence in a non‐pregnant premenopausal cohort with an adnexal mass (0.5%)^3^. The incidence of cyst resolution (24.0%) was lower than that reported by Condous *et al*. (71.7%) and Yazbek *et al*. (84.8%), which may be explained by our exclusion of physiological cysts and the later timing of the inclusion ultrasound scan, when cysts may have already resolved^1^, ^2^.

The incidence of malignancy (11/267 (4.1%)) was higher than that reported in low‐risk unselected pregnant populations described by Condous *et al*. (1/161 (0.62%)) and Yazbek *et al*. (0/728 (0%))^1^, ^2^. If we exclude women referred from local obstetric units/primary care (9/267), the incidence of malignancy within our own early‐pregnancy population that was followed up to the postnatal period drops to 3.9% (10/258). There were no suspected cases of invasive malignancy, which allowed for the deferral of surgical management to delivery or postnatal period to avoid the potential fetal and maternal risks associated with surgical intervention during pregnancy. In women with an adnexal mass referred to a regional oncology center, the incidence of malignancy (31.9%) and rate of antenatal surgical intervention (37.2%) were much higher, reflecting their high‐risk pregnant population^4^.

### Strengths and limitations

This is the first study to evaluate the use of BDs and the ADNEX model (where BDs cannot be applied) in a two‐step strategy to classify adnexal masses identified in a pregnant population within a university teaching hospital. Ultrasound variables were recorded prospectively to allow the retrospective application of the two‐step strategy. However, the study was limited by the small number of malignant tumors (*n* = 11 (4.1%)) and the lack of invasive cancers in this cohort, so it was not sufficiently powered to assess the discriminatory performance of the ADNEX model. We must also acknowledge the potential sources of bias: first, differential verification from using two different reference standards, given that only a subset of adnexal masses underwent surgery and so had histology as an endpoint; and second, selection and partial verification bias, given that a proportion of outcomes could not be determined because women were lost to follow‐up.

### Conclusions

Where applicable, BDs should be the first‐line approach for classifying adnexal masses during pregnancy. We recommend using expert SA to classify adnexal masses during pregnancy when BDs cannot be applied, until the performance of the ADNEX model has been assessed in a sufficiently powered study. Most adnexal masses can be managed expectantly, given the low risk of cyst‐related complications and high likelihood of spontaneous resolution. A large multicenter prospective trial is required to evaluate the test performance of the two‐step strategy and validate the use of ultrasound‐based diagnostic models to classify adnexal masses identified during pregnancy.

## Supporting information


**Table S1** Ultrasound features of 25 adnexal masses classified as malignant by the Assessment of Different NEoplasias in the adneXa model but as benign according to the reference standard (false positives)
**Table S2** Ultrasound features of 19 suspected decidualized endometriomas during pregnancy, according to expert subjective assessment
**Figure S1** Case 123. Adnexal mass was classified as mucinous cystadenoma on antenatal ultrasound but confirmed as mucinous borderline ovarian tumor on postnatal histology. On antenatal ultrasound, the lesion was described as unilocular, with low‐level content, a maximum diameter of 83 mm and no solid components, acoustic shadows or ascites. Benign descriptor 4 could be applied to classify this adnexal mass.
**Figure S2** Case 213. Adnexal mass was classified as suspected decidualized endometrioma on antenatal ultrasound according to expert subjective assessment but resolved postnatally. On antenatal ultrasound, the lesion was described as a multilocular solid (two locules), with a maximum diameter of 46 mm, maximum diameter of solid component of 14 mm, irregular cyst walls, two papillary projections, internal vascularity and acoustic shadows; risk‐of‐malignancy score according to the Assessment of Different NEoplasias in the adneXa model was 5.3%.
**Figure S3** Case 146. Adnexal mass was classified as suspected decidualized endometrioma (benign) on antenatal ultrasound according to expert subjective assessment and had prolonged period of resolution of decidualization postnatally (17 months). On antenatal ultrasound, the lesion was described as a unilocular solid, with a maximum diameter of 53 mm, maximum diameter of solid component of 14 mm, irregular cyst walls, one papillary projection, no internal vascularity and no acoustic shadows; risk‐of‐malignancy score according to the Assessment of Different NEoplasias in the adneXa model was 24.3%.
**Figure S4** Case 231. Adnexal mass was classified as suspected decidualized endometrioma on antenatal ultrasound, as borderline ovarian tumor (BOT) on postnatal ultrasound and serous BOT on histology. On antenatal ultrasound, the lesion was described as a unilocular solid, with a maximum diameter of 19 mm, maximum diameter of solid component of 13 mm, irregular cyst walls, one papillary projection and internal vascularity; risk‐of‐malignancy score according to the Assessment of Different NEoplasias in the adneXa model was 34.3%.

## Data Availability

The data that support the findings of this study are not available due to ethical restrictions of sharing patient data.
