# Supplementary material for: Evaluating use of two‐step International Ovarian Tumor Analysis strategy to classify adnexal masses identified in pregnancy: pilot study
Source: Ultrasound Obstet Gynecol. 2024 Dec 2;64(6):808–17. doi: 10.1002/uog.27707 (PMC11609963; doi:10.1002/uog.27707)
Supplement: Supplementary file 1 — Table S1 Ultrasound features of 25 adnexal masses classified as malignant by the Assessment of Different NEoplasias in the adneXa model but as benign according to the reference standard (false positives) Table S2 Ultrasound features of 19 suspected decidualized endometriomas during pregnancy, according to expert subjective assessment Figure S1 Case 123. Adnexal mass was classified as mucinous cystadenoma on antenatal ultrasound but confirmed as mucinous borderline ovarian tumor on postnatal histology. On antenatal ultrasound, the lesion was described as unilocular, with low‐level content, a maximum diameter of 83 mm and no solid components, acoustic shadows or ascites. Benign descriptor 4 could be applied to classify this adnexal mass. Figure S2 Case 213. Adnexal mass was classified as suspected decidualized endometrioma on antenatal ultrasound according to expert subjective assessment but resolved postnatally. On antenatal ultrasound, the lesion was described as a multilocular solid (two locules), with a maximum diameter of 46 mm, maximum diameter of solid component of 14 mm, irregular cyst walls, two papillary projections, internal vascularity and acoustic shadows; risk‐of‐malignancy score according to the Assessment of Different NEoplasias in the adneXa model was 5.3%. Figure S3 Case 146. Adnexal mass was classified as suspected decidualized endometrioma (benign) on antenatal ultrasound according to expert subjective assessment and had prolonged period of resolution of decidualization postnatally (17 months). On antenatal ultrasound, the lesion was described as a unilocular solid, with a maximum diameter of 53 mm, maximum diameter of solid component of 14 mm, irregular cyst walls, one papillary projection, no internal vascularity and no acoustic shadows; risk‐of‐malignancy score according to the Assessment of Different NEoplasias in the adneXa model was 24.3%. Figure S4 Case 231. Adnexal mass was classified as suspected decidualized endometrioma on antenatal ultr [file UOG-64-808-s001.docx]

**Table S1:** Ultrasound features of 25 adnexal masses classified as malignant by the Assessment of Different NEoplasias in the adneXa (ADNEX) model but as benign according to the reference standard (false positives)

| **Case ID** | **External referral**  **(Y/N)** | **Max**  **lesion size (mm)** | **Gestation at USS assessment** | **Lesion type** | **Locules** | **Number of Papillary projections** | **Max solid component (mm)** | **Acoustic shadows (Y/N)** | **Ascites (Y/N)** | **ADNEX SCORE**  **(Risk of malignancy, without CA125, %)** | **Diagnosis according to expert SA or histology** |
| --- | --- | --- | --- | --- | --- | --- | --- | --- | --- | --- | --- |
| *2* | N | 76 | *18* | *Unilocular solid* | 1 | 1 | **23** | **N** | N | 35.2 | Mucinous cystadenoma (histology) |
| *16* | N | 102 | *22* | *Unilocular solid* | 1 | 2 | **33** | **N** | **N** | 54.1 | Decidualised endometrioma (expert SA) |
| *35* | N | 18 | *14* | *Unilocular solid* | 1 | 1 | **6** | **N** | **N** | 14.6 | Cystadenofibroma (expert SA) |
| *42* | N | 33 | *12* | *Unilocular solid* | 1 | 1 | **21** | **N** | **N** | 41.2 | Decidualised endometrioma (expert SA) |
| *62* | N | 54 | *8* | *Unilocular solid* | 1 | 1 | **14** | **N** | **N** | 23.3 | Decidualised endometrioma (expert SA) |
| *106* | N | 17 | *13* | *Unilocular solid* | 1 | 1 | **5** | **N** | **N** | 14.3 | Benign Corpus Albicans (histology) |
| *118* | N | 29 | *14* | *Unilocular solid* | 1 | 1 | **14** | **N** | **N** | 30.1 | Decidualised endometrioma (expert SA) |
| *136* | N | 18 | *14* | *Unilocular solid* | 1 | 1 | **6** | **N** | **N** | 14.6 | Cystadenofibroma (expert SA) |
| *146* | N | 53 | *8* | *Unilocular solid* | 1 | 1 | **14** | **N** | **N** | 24.3 | Decidualised endometrioma (expert SA) |
| *169* | N | 45 | *20* | *Unilocular solid* | 1 | 1 | **20** | **N** | **N** | 24.5 | Benign cystadenoma (expert SA) |
| *175* | N | 35 | *26* | *Unilocular solid* | 1 | 0 | **16** | **N** | **N** | 24.3 | Benign cystadenoma (expert SA) |
| *185* | N | 50 | *16* | *Unilocular solid* | 1 | 1 | **9** | **N** | **N** | 14.3 | Benign cystadenoma (expert SA) |
| *196* | N | 17 | *8* | *Unilocular solid* | 1 | 1 | **11** | **N** | **N** | 29.3 | Decidualised endometrioma (expert SA) |
| *205* | N | 48 | *17* | *Unilocular solid* | 1 | 1 | **11** | **N** | **N** | 18.3 | Decidualised endometrioma (expert SA) |
| *211* | N | 35 | *9* | *Unilocular solid* | 1 | 2 | **6** | **N** | **N** | 17.1 | Paraovarian cyst (expert SA) |
| *221* | N | 56 | *26* | *Multilocular solid* | 3 | 0 | **11** | **N** | **N** | 10.4 | Cystadenofibroma |
| *224* | N | 45 | *17* | *Unilocular solid* | 1 | 1 | **11** | **N** | **N** | 18.6 | Decidualised endometrioma (expert SA) |
| *233* | N | 110 | *20* | *Multilocular solid* | *2* | 0 | **21** | **N** | **N** | 18.7 | Mucinous Cystadenoma (histology) |
| *241* | N | 34 | *19* | *Unilocular solid* | 1 | > 4 | **21** | **N** | **N** | 75.0 | Decidualised endometrioma (expert SA) |
| *246* | N | 73 | *19* | *Unilocular solid* | 1 | 2 | **24** | **N** | N | 45.2 | Cystadenofibroma/dermoid (expert SA) |
| *251* | N | 52 | *21* | *Solid* | NA | 0 | **52** | **N** | N | 51.0 | Decidualised endometrioma (expert SA) |
| *254* | N | 36 | *13* | *Multilocular solid* | >10 | 1 | **21** | **N** | N | 65.6 | Cystadenofibroma (expert SA) |
| *266* | N | 63 | *7* | *Multilocular solid* | 2 | 1 | **6** | N | N | 11.4 | Decidualised endometrioma (expert SA) |
| *277* | N | 40 | *14* | *Unilocular solid* | 1 | 3 | **12** | N | N | 46.1 | Decidualised endometrioma (expert SA) |
| *287* | N | 42 | *9* | *Unilocular solid* | 1 | 1 | 13 | N | N | 23.2 | Decidualised endometrioma (expert SA) |

Y= yes, N=no

**Table S2:** Ultrasound features of 19 suspected decidualized endometriomas during pregnancy, according to expert subjective assessment

| ***Case ID*** | ***Gestation*** | ***Max***  ***lesion size (mm)*** | ***Lesion type*** | ***Locules*** | ***Irregular cyst wall (Y/N)*** | ***Colour score*** | ***Number of papillary projections*** | ***Papillary flow (Y/N/NA)*** | ***Max solid component (mm)*** | ***Acoustic shadows (Y/N)*** | ***ADNEX SCORE***  ***(Risk of malignancy no CA125)*** | ***Postnatal (PN) outcome*** | ***USS pre pregnancy (Y/N)*** | ***Postnatal USS (months)*** | ***Referral origin*** |
| --- | --- | --- | --- | --- | --- | --- | --- | --- | --- | --- | --- | --- | --- | --- | --- |
| *16* | *22* | 102 | *Unilocular solid* | *1* | Y | 3 | 2 | Y | **33** | **N** | 54.1 | Endometrioma | Y | 2 | 1 |
| *42* | *12* | 33 | *Unilocular solid* | *1* | Y | 1 | 1 | N | **21** | **N** | 41.2 | Endometrioma | Y | 3 | 1 |
| *56* | *14* | 37 | *Unilocular* | *1* | N | 1 | 0 | NA | **0** | **N** | 2.1 | Endometrioma | Y | 2 | 2 |
| *62* | *8* | 54 | *Unilocular solid* | *1* | Y | 1 | 1 | N | **14** | **N** | 23.3 | Endometrioma | Y | 13 | 2 |
| *109* | *8* | 31 | *Unilocular* | *1* | Y | 2 | 0 | NA | **0** | **N** | 1.9 | Endometrioma | N | 2 | 2 |
| *118* | *14* | 29 | *Unilocular solid* | *1* | Y | 3 | 1 | Y | **14** | **N** | 30.1 | Endometrioma | Y | 9 | 2 |
| *124* | *12* | 73 | *Multilocular solid* | *2* | Y | 2 | 1 | N | **8** | **Y** | 1.6 | Endometrioma | Y | 6 | 2 |
| *146* | *8* | 53 | *Unilocular solid* | *1* | Y | 2 | 1 | N | **14** | **N** | 24.3 | Resolving decidualised endometrioma  (17 months PN) | Y | 1 | 2 |
| *159* | *8* | 67 | *Unilocular* | *1* | Y | 2 | 0 | NA | **0** | **N** | 3.8 | Endometrioma | Y | 1.5 | 2 |
| *196* | *8* | 17 | *Unilocular solid* | *1* | Y | 2 | 1 | N | **11** | **N** | 29.3 | Endometrioma | N | 2 | 2 |
| *205* | *17* | 48 | *Unilocular solid* | *1* | Y | 2 | 1 | Y | **11** | **N** | 18.3 | Endometrioma | Y | 2 | 2 |
| *213* | *20* | 46 | *Multilocular solid* | *2* | Y | 2 | 2 | Y | **14** | **Y** | 5.3 | Endometrioma | N | 2 | 1 |
| *224* | *17* | 45 | *Unilocular solid* | *1* | Y | 1 | 1 | Y | **11** | **N** | 18.6 | Endometrioma | Y | 4 | 2 |
| *231* | *13* | 19 | *Unilocular solid* | *1* | Y | 1 | 1 | Y | **13** | **N** | 34.3 | Serous BOT | N | 4 | 2 |
| *241* | *19* | 34 | *Unilocular solid* | *1* | Y | 4 | >4 | Y | **21** | **N** | 75.0 | Resolved | Y | 4 | 1 |
| *251* | *21* | 52 | *Solid* | *0* | Y | 1 | 0 | NA | **52** | **N** | 51 | Endometrioma | N | 3 | 2 |
| *266* | *7* | 63 | *Multilocular solid* | *2* | Y | 2 | 1 | N | **6** | N | 11.4 | Endometrioma | Y | 1 | 2 |
| *277* | *14* | 40 | *Unilocular solid* | *1* | Y | 4 | 3 | Y | **12** | N | 46.1 | Endometrioma | N | 3 | 1 |
| *287* | *19* | 42 | *Unilocular solid* | *1* | Y | 3 | 1 | Y | **13** | N | 23.2 | Endometrioma | N | 14 | 2 |

Y= yes, N=no

**Supplementary Figures**

**Figure S1:** Case 123. Adnexal mass was classified as mucinous cystadenoma on antenatal ultrasound but confirmed as mucinous borderline ovarian tumor on postnatal histology. On antenatal ultrasound, lesion was described as unilocular, with low level content, a maximum diameter of 83 mm and no solid components, acoustic shadows or ascites. Benign descriptor 4 could be applied to classify this adnexal mass.


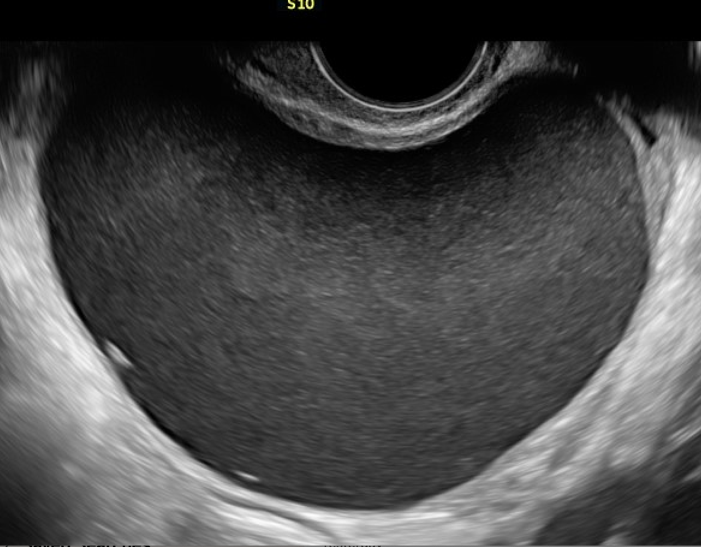


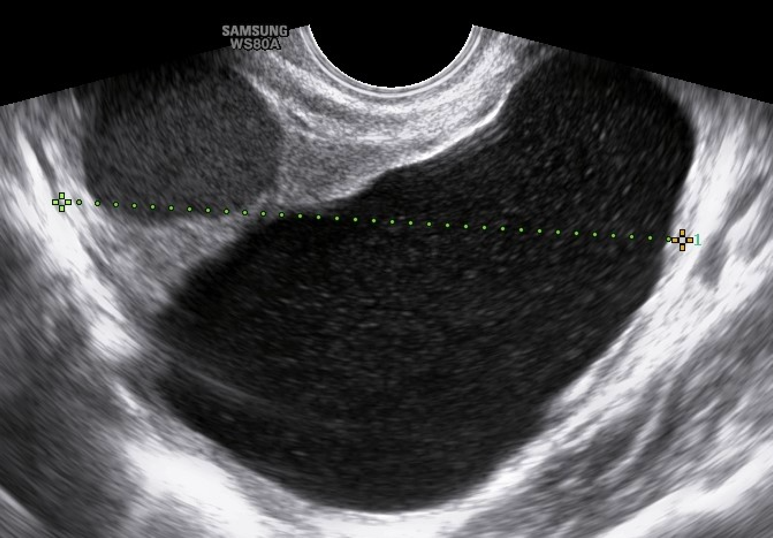


15 weeks 2 months PN

**Figure S2:** Case 213. Adnexal mass was classified as suspected decidualized endometrioma on antenatal ultrasound according to expert subjective assessment but resolved postnatally. On antenatal ultrasound, the lesion was described as multilocular solid (two locules), with a maximum diameter of 46 mm, maximum diameter of solid component of 14 mm, irregular cyst walls, two papillary projections, internal vascularity and acoustic shadows; risk of malignancy score according to Assessment of Different NEoplasias in the adneXa (ADNEX) model was 5.3%.

***
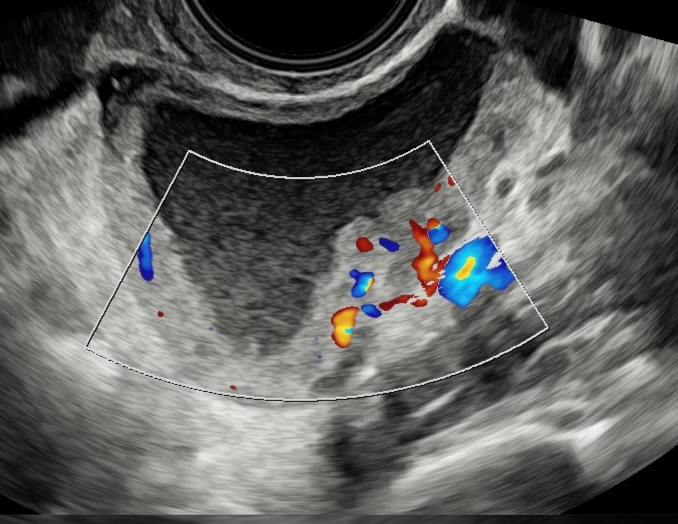
***
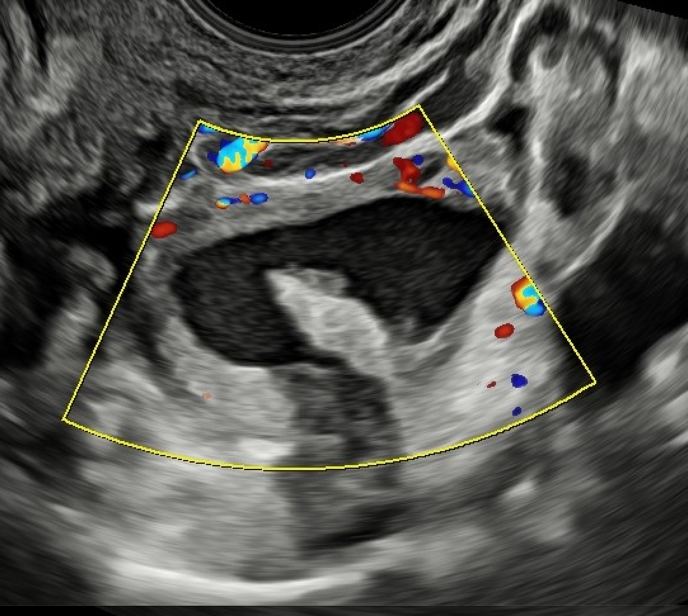

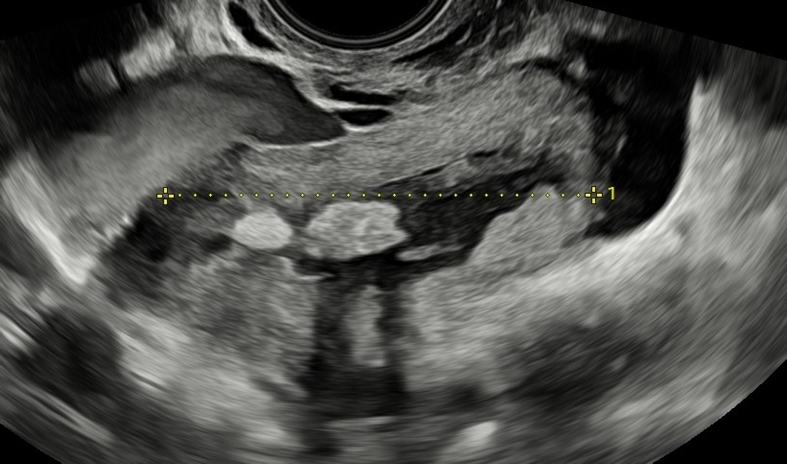


21 weeks 24 weeks 6 months PN

**Figure S3:** Case 146. Adnexal mass was classified as suspected decidualized endometrioma (benign) on antenatal ultrasound according to expert subjective assessment and had prolonged period of resolution of decidualization postnatally (17 months). On antenatal ultrasound, the lesion was described as unilocular solid, with a maximum diameter of 53 mm, maximum diameter of solid component of 14 mm, irregular cyst walls, one papillary projection, no internal vascularity and no acoustic shadows; risk of malignancy score according to Assessment of Different NEoplasias in the adneXa (ADNEX) model was 24.3%.

***
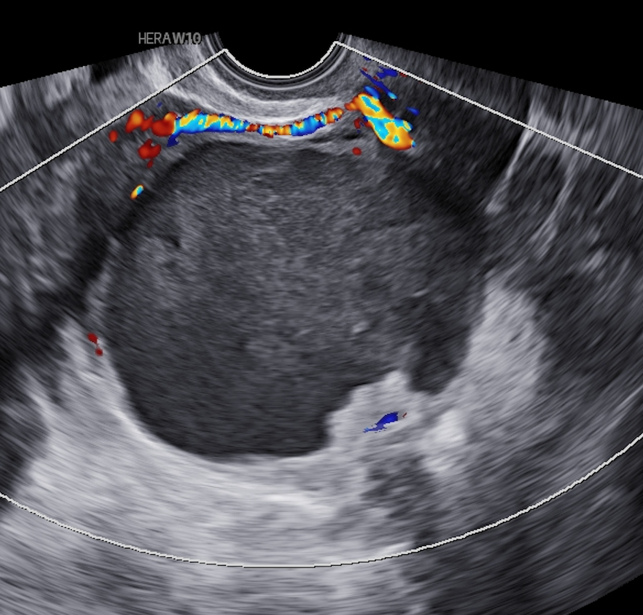
***
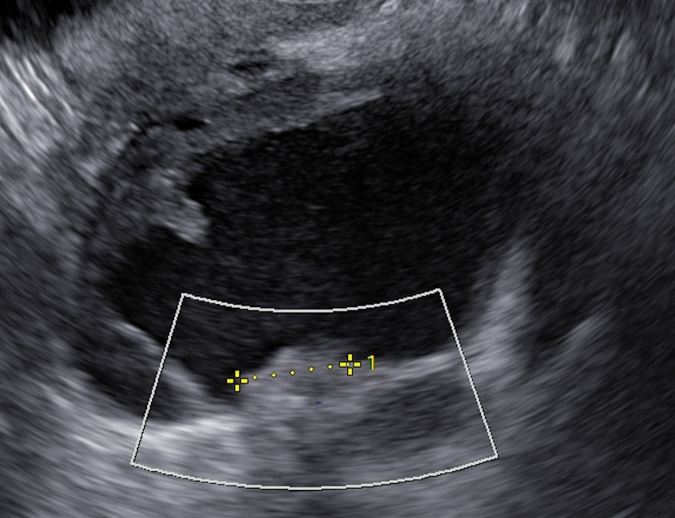

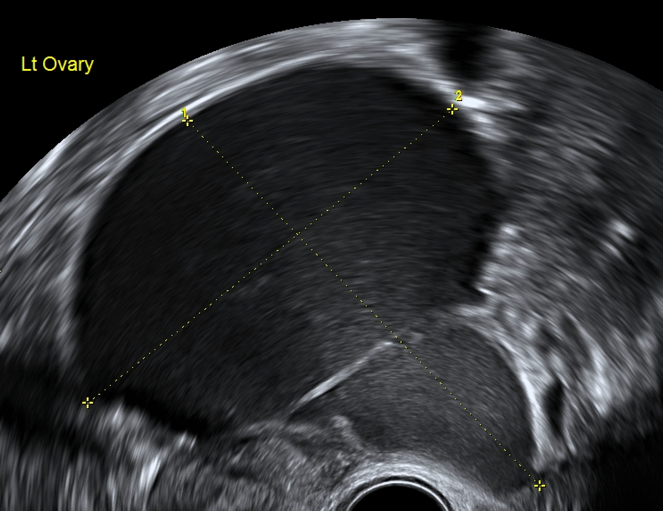

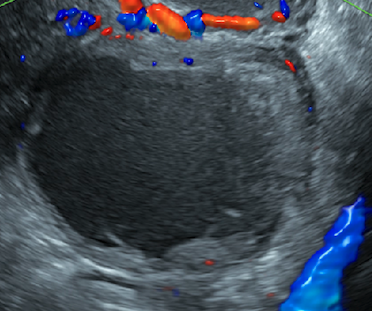


8 weeks pregnant 1 month Postnatal 8 month Postnatal 17 month Postnatal

**Figure S4:** Case 231. Adnexal mass was classified as suspected decidualized endometrioma on antenatal ultrasound, as borderline ovarian tumor (BOT) on postnatal ultrasound and serous BOT on histology. On antenatal ultrasound, lesion was described as unilocular solid, with a maximum diameter of 19 mm, maximum diameter of solid component of 13 mm, irregular cyst walls, one papillary projection and internal vascularity; risk of malignancy score according to Assessment of Different NEoplasias in the adneXa (ADNEX) model was 34.3%.

*
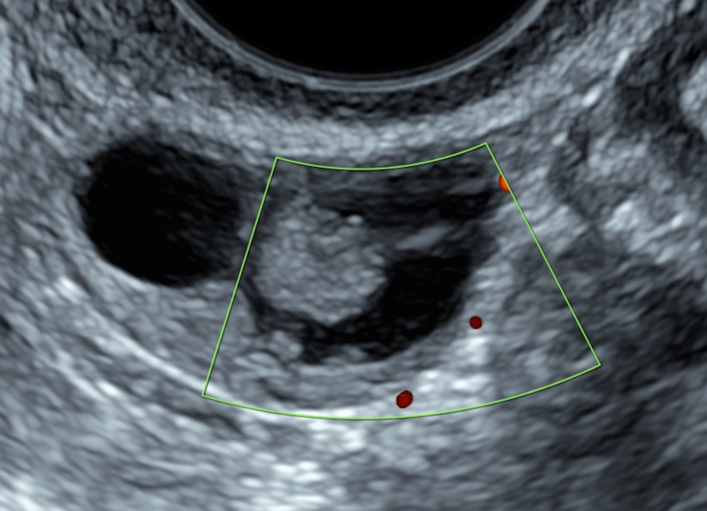

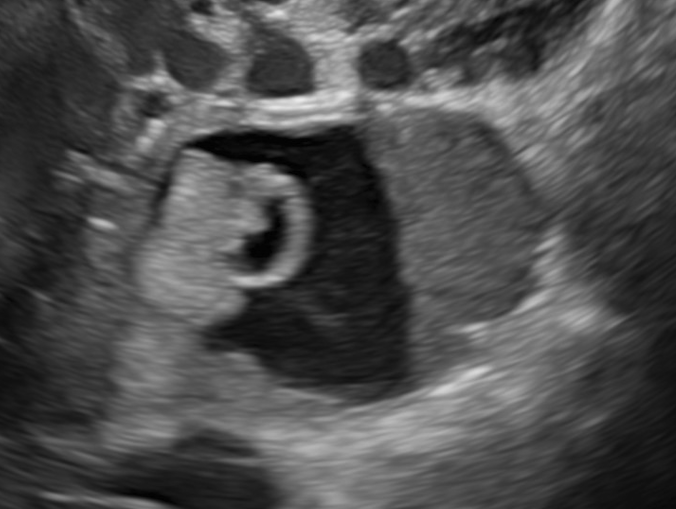
*

*21 weeks 4 months PN*
